# An approach to assess stress in response to drive hunts using cortisol levels of wild boar (*Sus scrofa*)

**DOI:** 10.1038/s41598-021-95927-2

**Published:** 2021-08-12

**Authors:** Justine Güldenpfennig, Marion Schmicke, Martina Hoedemaker, Ursula Siebert, Oliver Keuling

**Affiliations:** 1 Institute for Terrestrial and Aquatic Wildlife Research, University of Veterinary Medicine Hannover, Foundation, Bischofsholer Damm 15, 30173 Hannover, Germany; 2 Institute of Agricultural and Food Sciences, Animal Health Management, Martin Luther University Halle-Wittenberg, Theodor-Lieser-Straße 11, 06120 Halle, Germany; 3 Clinic for Cattle, University of Veterinary Medicine Hannover, Foundation, Bischofsholer Damm 15, 30173 Hannover, Germany; 4 Department of Game Management and Wildlife Biology, Faculty of Forestry and Wood Sciences, Czech University of Life Sciences Prague, 165 21 Prague, Czech Republic

**Keywords:** Physiology, Zoology, Ecology, Behavioural ecology, Ecophysiology

## Abstract

Hunting can easily be linked to stress in wildlife. Drive hunts performed two to three times in one area during the respective hunting period, are thought to decrease the pressure hunting places on wildlife. Nevertheless, the expression of cortisol—one of the main mammalian stress hormones—is considered to have negative impacts on animals’ well-being if expressed excessively, which may occur during some (especially repeated) hunting events. We explored the effect of drive hunts on cortisol levels in wild boar in Lower Saxony, Germany, compared these cortisol levels to reference values given by a similar study, and investigated the effect of age, sex, and pregnancy. Blood collected from wild boar shot on drive hunts was analysed using a radioimmunoassay. As expected, we observed elevated cortisol levels in all samples, however, we still found significant differences between age groups and sexes, as well as an influence of pregnancy on cortisol levels. The effect of drive hunts on cortisol levels appears to be weaker than predicted, while the effects of other variables, such as sex, are distinct. Only half of the evaluated samples showed explicitly increased cortisol levels and no significant differences were found between sampling months and locations. Group living animals and pregnant females showed significantly higher cortisol levels. The impact of hunting is measurable but is masked by natural effects such as pregnancy. Thus, we need more information on stress levels in game species.

## Introduction

Interest in stress research based on animal welfare is growing^1^, given the fact that stress, especially long term, can negatively affect an animal’s well-being^2–4^. Stress is a widely used term in human society and could be defined as “the nonspecific response of the body to any demand of change”^5,6^. In general, stress is a change in the psychological, physiological, and/or physical features of an organism^7^. During stress, the animal’s behaviour is altered to enhance attention, increase cardiac output, respiration, and catabolism, as well as to divert blood flow to provide full perfusion of the brain, heart, and muscles^8^. Responding to a stressor, the hypothalamus–pituitary–adrenal (HPA) axis is activated, which results in the secretion of its end hormones, the glucocorticoids. One of those glucocorticoids and the most common stress hormone in mammals, cortisol, plays a key role in energy release, immune and mental activity, development, and growth, as well as in reproductive functions^9,10^. In its important role in stress response, cortisol is sensitive to both physical and emotional stimuli; the release underlies a circadian rhythm^9^. Under physiologic stress, cortisol modulates the immune system and mobilizes energy storage, making more resources available for responding to a certain stressor^10,11^. While glucocorticoids are beneficial for short-term survival, enduring/increased release (chronic stress) can lead to metabolic, immune, and physiological dysfunction^2^. Therefore, the cumulative occurrence of stressors could lead to changes in an animal’s well-being and changes in social and disease networks^3,12^. It was found that chronic stress in domestic pigs leads to altered cortisol levels, reduced growth, and reduced play-behaviour^13^. High cortisol levels are also linked to many behavioural, physiological, nutritional diseases and disorders^7^, as well as to obesity and diabetes caused by increasing plasma glucose concentrations due to cortisol^14^. Therefore, a better understanding of stress and its effect on organisms seems to be necessary in not only humans and livestock, but also in wildlife. Despite the negative effects on the well-being, stress and high cortisol levels are most likely to influence the relationship between wildlife and disease as well as host-parasite equilibrium, which can influence animal populations and lead to loss of biodiversity^7^. Causes for stress in wildlife could be caused by natural events, e.g. the elements and insufficient food and/or water resources, as well as human-caused events, e.g. deforestation, habitat fragmentation, as well as human disturbances such as tourism and hunting^7,15^.

The wild boar *Sus scrofa* (Linnaeus, 1758) is nowadays distributed across almost all continents and shows one of the most widely spread native geographic ranges^16–18^. Because of some traits, such as opportunistic feeding and the diversity of habitats they occupy^17^, they may not seem to be the best model organism for stress research, assuming relatively low-stress responses to chronic stress triggers such as food limitation and habitat loss. However, blood samples of wild boar are easy to collect in large numbers due to high-density populations in almost every habitat^17^ and an all-year hunting season^19^. Besides, behavioural and social organizations in this species are well known^17,18^, allowing for the investigation of stress differences between sexes, ages, and social positions. Only a few studies about cortisol in wild boar are available, which show differences in sampling method (e.g. sampling from living animals with trapping^20^ or from dead animals after hunting and other trauma^21^) and other limitations^20^. Differences between sampling method and hormonal assays used make it difficult to compare cortisol levels itself. Gentsch et al. defined “normal” (shot on single hunts, no previous disturbance) and “trauma” (shot on driven hunts with previous disturbance, death due to accidents) cortisol levels of wild boar and other ungulate species in response to different hunting methods^21^.

In other pig species and domestic pigs, there are said to be differences between the responses to chronic stress of males and females, as well as different social ranks in pigs^22^. Knowledge of wild boar stress and stressors may also help to improve domestic pigs’ welfare in pig farming, by supplementing the available stress research specific to domestic pigs.

In the last decades, wild boar populations are increasing, which could lead to different economic problems such as damage to crop fields and forests due to their foraging behaviour^23–25^. Another more recent problem is the probability of wild boar transmitting diseases on livestock^26,27^. To control the wild boar population under the aspect of disease control, the hunting intensity increased, e.g. currently because of the African swine fever^28^. Properly performed single hunts are often not sufficient to achieve a huge reduction of wild boar populations. They are time-consuming and assumed to resolve in more stress for wildlife due to the repeating presence of the hunter in their environment^29,30^. Linked to a decreased hunting pressure because of the concentration on 1 or 2 days, drive hunts are considered to reduce hunting pressure-induced stress^29–31^, although other approaches state that drive hunts increase stress^21,32^.

There is a variety of biological substances to measure cortisol levels non-invasively, such as saliva, faeces, and hair^1,33^. Although salivary cortisol levels have been considered to be a better way to determine stress in animals, these data have to be treated with caution, as saliva contains only biologically free cortisol which does not respond linearly to a challenge^34^. Also, collecting saliva from free-ranging animals requires some kind of collecting device and collecting from dead animals remains challenging, as blood contaminates snouts of shot animals after death, which has to be avoided^33^. Faeces only contain metabolites of cortisol from the past hours to days, based on metabolism^35^. A much longer record of cortisol levels can be measured using hair, which makes it useful in measuring chronic stress^33^. The most widely used method to evaluate stress in captive animals is measuring serum cortisol levels. Only 15–30 min. after the exposition to a stressor or after handling, serum cortisol levels peak, and basal levels are reobtained after approximately 60–90 min^21,36^. A sampling of blood in free-ranging animals often includes hunting (to sample from dead animals, often possible in larger quantities) or capturing and handling or, which can be considered as additional stressors and could lead to elevated cortisol values. To measure basal stress levels, this could lead to a distortion of cortisol values obtained from wildlife. Nonetheless, we used blood serum to determine cortisol during drive hunts (at the time of death), as it the most useful while measuring acute stress during hunting, and with other methodological advantages, e.g. sampling.

Since there is a need for a better understanding of stress mechanisms in wildlife, and in this case under hunting pressure, this work serves as a pilot study to measure stress in wildlife and investigate the effect of drive hunts on the cortisol levels, and therefore on the acute stress, of intensively hunted game species such as the wild boar in Lower Saxony. We investigated the following questions: (1) What are the stress levels of wild boar during drive hunts (measured as blood cortisol levels as blood taken from wild boar after the hunt), (2) can we ascertain elevated cortisol levels caused by stressors such as repeated drive hunts using values given in a similar study^21^, and (3) are there differences between age groups and sexes, as well as pregnant and non-pregnant wild boar? Pregnancy, as another factor possible to influence cortisol levels^37,38^, should be taken into account to exclude its effect on possible differences between the stress response of male and female wild boar. Additionally, we investigated the effect of weight on cortisol levels, as metabolism was recently linked to cortisol^4^.

## Materials and methods

### Study area

The study area was located in the eastern part of the federal state of Lower Saxony (Northern Germany) (52.36° N, 10.35° E) (similar to Refs.^39,40^). Altitude ranged from 60 to 130 m above sea level with subcontinental climates^41^. The average annual precipitation and the average annual temperature in 2018 were 512 mm and 10.7 °C, respectively^42^. The approximately 4900 km^2^ area was composed of 54.6% of cultivated fields and 27.6% forest, with buildings and infrastructure claiming the remaining 17.8% (for detailed information see^39,40^). In the study area, hunting was usually performed as a single hunt or a drive hunt. Wild boar hunting has especially intensified (increased hunting bag and other measures) due to risk control management associated with African swine fever^28^. Drive hunts were usually performed between November–January, and one or two times in the same area during the hunting period. The effect of possible differences such as hunting intensity (although expected to be non-existent or very low), habitat structure (topography, wet parts, area surrounded by forest/agriculture/…), and microclimate on cortisol levels between the respective forestry offices was excluded while comparing cortisol levels between each hunting district. Hunting districts are defined as separate areas within the domain of the forestry office. Each hunting district is under the jurisdiction of lower forestry offices who manage among others all hunting activities in this area. Each hunting district varies slightly in their habitat composition, mainly in forest-field proportion, altitude, or amount of wet parts. This could influence microclimate and behaviour of wild boar, e.g. more forest areas allow more efficient hiding, more wet parts complicate moving for both wild boar and hunters.

### Sample collection and laboratory analysis

We collected blood from ~ 300 shot wild boar after drive hunts in the western part of Lower Saxony, Germany. The blood was collected using 7.5 mL KABEVETTES (Kabe Labortechnik GmbH, Nümbrecht-Elsenroth) with serum coagulation inducer (granules and kaolin) during slaughtering, max. 3 h after death, by cutting the jugular vein or other large blood vessels in the chest cavity. The blood samples were stored in a cold box until arriving back at the institute (2–4 h after sampling). We centrifuged the blood samples for 12 min. at 4500 rpm, transferred 1 mL serum to 1.5 mL Eppendorf Tubes (Eppendorf AG, Hamburg), and stored them at − 32 °C until assay. The shot animals were categorized in age classes based on their tooth eruption^41^. We classified animals as j = juveniles < 12 months (piglets), y = yearlings 12–23 months or a = adults ≥ 24 months, as well as differentiating between the two sexes f = females and m = males^43^. In total, we chose 115 samples for examination with similar sample size in all age-sex classes (mj = 22, fj = 28, my = 20, fy = 14, ma = 16, fa = 15). The effect of haemolysis on the hormonal assay with this kit was unknown and therefore homolysed samples were excluded. This subsample contained of samples from October/November (n = 62), December (n = 20) and January (n = 33), with 57 samples from female and 58 samples from male wild boar. October was included in November because of the small sample size. Hunts were only performed in the last week of October.

Laboratory analysis was conducted at the Endocrinological Laboratory of the Clinic for Cattle of the University of Veterinary Medicine Hannover, Foundation using radioimmunoassay (Cortisol RIA KIT, Beckmann Coulter, Inc., Krefeld) while following the instructions from the manufacturer. Additionally, we sampled uteri and ovaries for the ongoing analysis of the reproductive status of female wild boar (according to the procedures of Refs.^39,40,44^). With this information, we categorized female wild boar as potentially pregnant (several corpora lutea, but no visible embryos; n = 7), pregnant (developed fetuses; n = 14), and non-pregnant (no visible corpora lutea or fetuses; n = 26).

We compared the distribution of our values to reference values given by Gentsch et al., which set 249.6 ± 36.8 nmol/L as normal and 775.2 ± 64.7 nmol/L as trauma cortisol levels in wild boar^21^. Using these values, we estimated the effect of drive hunts on stress.

### Ethical statement

We did not conduct animal experiments in the meaning of German legislation. Thus, all experiments were carried out in compliance with ARRIVE guidelines. All described sampling methods were conducted during or after normal legal hunting activities due to the laws of the Federal Republic of Germany and the Federal State of Lower Saxony and all international and/or institutional guidelines for animal handling were followed. No animals were harmed or killed for our sampling specifically. No licenses were needed for animal testing due to German legislation sampling dead animals. Sampling allowances were given by the forestry commission offices Oerrel, Unterlüß and Wolfenbüttel as well as the “Verwaltung Günther Graf v. d. Schulenburg”. No ethical permit for animal experiments applies or must be permitted. This study did not use human participants.

### Statistical analysis

All statistics were done with R version 3.5.2^45^. We tested for significant differences using Kruskal–Wallis analysis of variance between cortisol level means by sampling month, hunting grounds, state of pregnancy in female wild boar, age group, and sex, as well as by age and sex combined (classification in agesex groups “male juvenile”, “female juvenile”, “male yearlings”, “female yearlings”, “male adults”, and “female adults”). Post-hoc analysis was performed using Dunn’s Kruskal–Wallis multiple comparison test^46^ with the FSA package^47^, with p-values adjusted using the Benjamini–Hochberg method. The confidence level was set as α = 0.05. Possible correlation between weight and cortisol was tested using the ggpubr package^48^.

## Results

**Figure 1:**
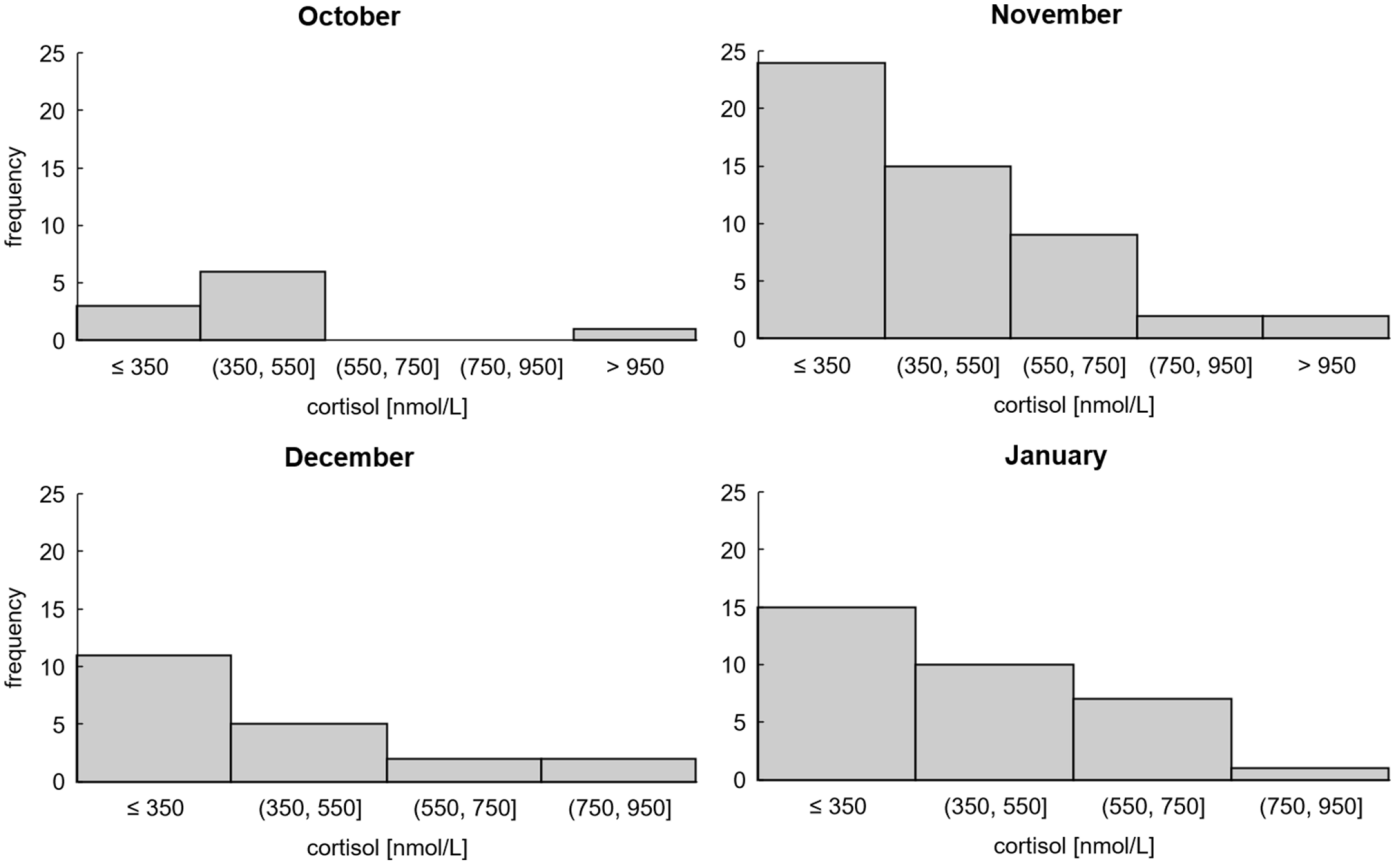
Cortisol levels of wild boar clustered in blocks matching Gentsch et al. Clusters of cortisol levels below 350 nmol/L are defined as normal/baseline levels and anything above as increased trauma levels caused by increased stress during drive hunts and compared the hunting months October (n = 10), November (n = 52), December (n = 20) and January (n = 33). There is no difference between hunting months.

Viewing our results in clusters comparable to levels found by Gentsch et al., it is notable that only 54% of our cortisol values could be considered as increased trauma levels (> 350 nmol/L), whereas 38% of our values could be defined as normal cortisol levels (150–350 nmol/L). The remaining 8% are out of range. The mean cortisol level and standard deviation of the total sample is 411.2 ± 242.6 nmol/L; the median is 364.7 nmol/L. Minimum and maximum cortisol levels are 30.6 nmol/L and 1457.9 nmol/L, respectively. The mean cortisol levels ± standard deviation of animals shot in October/November, December, and January are 419.2 ± 278.7 nmol/L, 383.5 ± 236.7 nmol/L, and 412.9 ± 166.2 nmol/L, respectively (Fig. 1). Although our values show small differences, there are no significant differences among months (Kruskal–Wallis chi-squared = 0.96165, df = 2, p-value = 0.6183).

**Figure 2:**
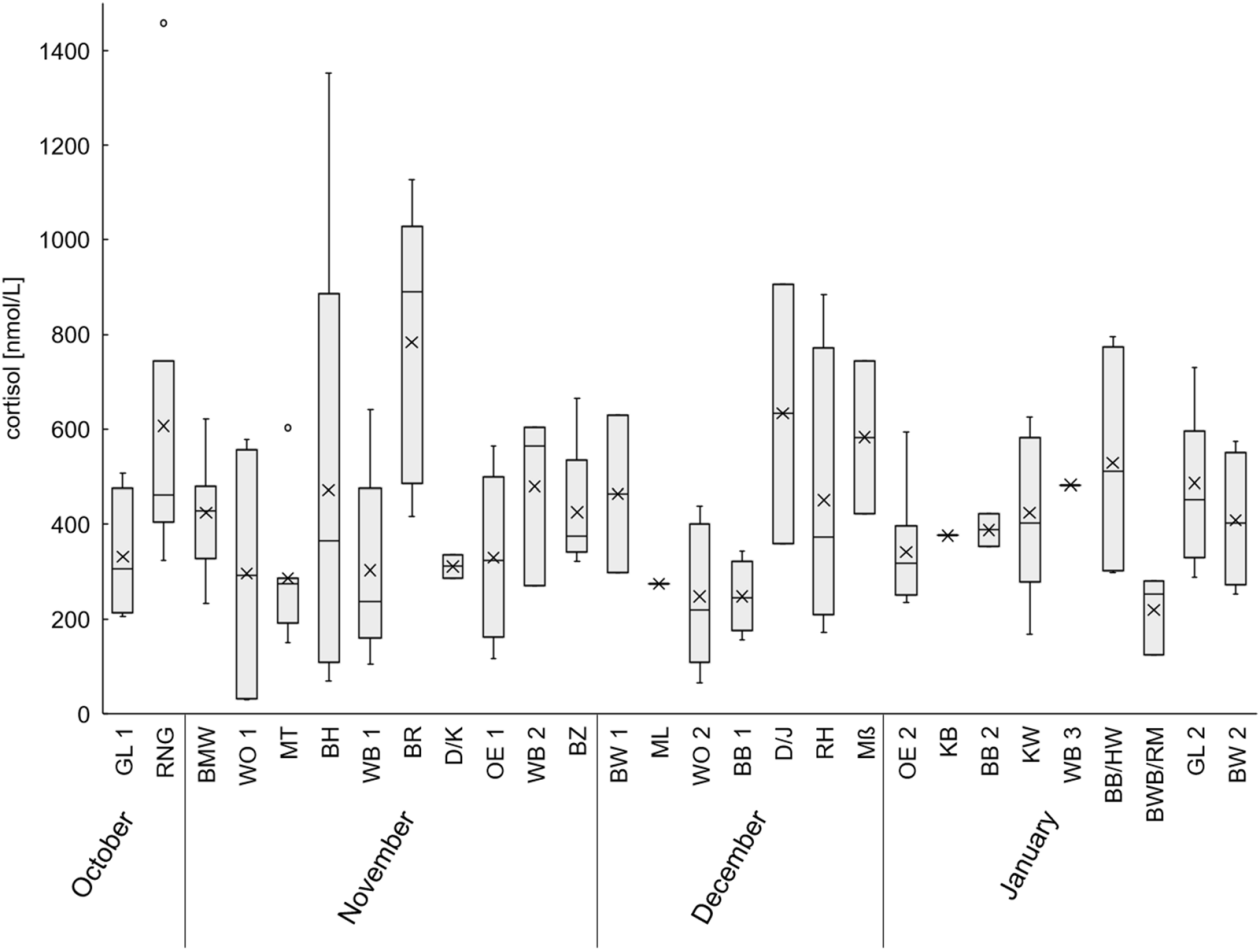
Cortisol levels of wild boar compared among evaluated hunting grounds. The sampling locations are sorted chronologically from October until January. The abbreviations serve as an orientation for the different hunting grounds, which will not be named further because of the protection of privacy.

The mean cortisol levels show high variation between the different hunting districts (Fig. 2). However, due to the small sample size, there are no significant differences between hunting districts (Kruskal–Wallis chi-squared = 35.701, df = 27, p-value = 0.122).

**Figure 3:**
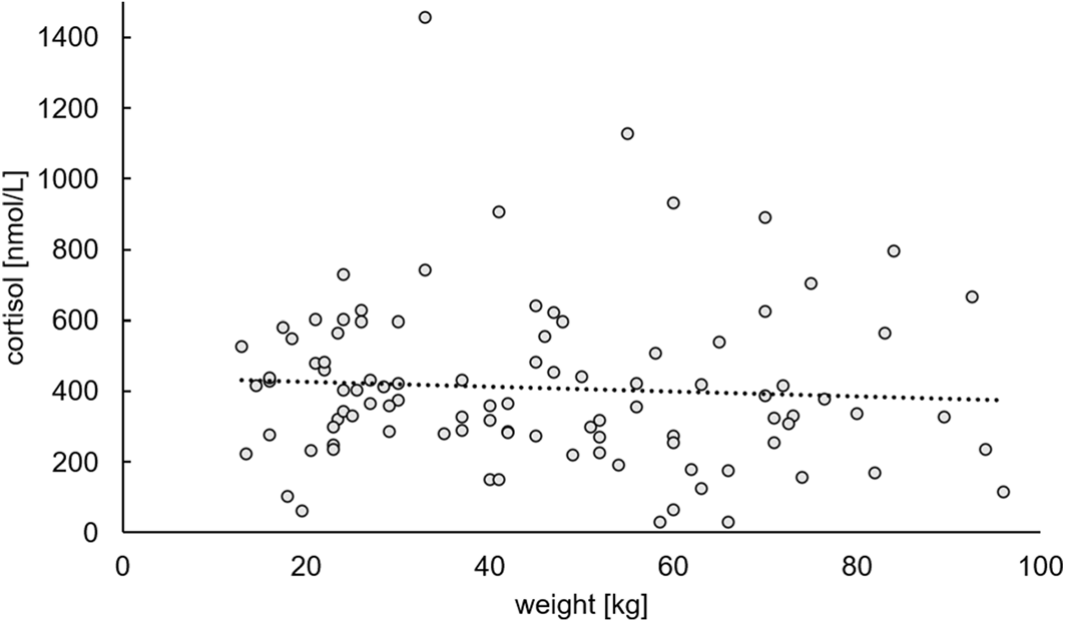
Correlation between cortisol levels and weight of wild boar. The values are highly variable, and no correlation could be found. Regression line: y =  − 0.6801x + 440.79; R^2^ = 0.0042.

We additionally tested for correlation between cortisol levels and dressed weight (weight after gutting) but did not find any correlation (Pearson’s product-moment correlation: t =  − 0.63456, df = 96, p-value = 0.5272, cor =  − 0.06462947; Fig. 3).

**Figure 4:**
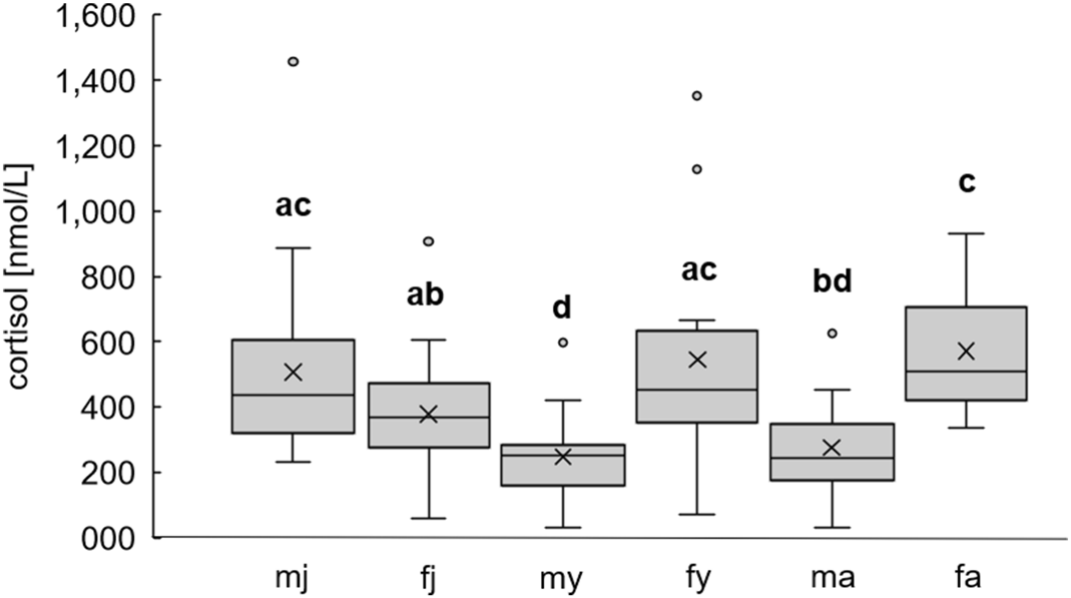
Differences of cortisol levels of wild boar between each age class and sex. Female wild boar (f, n = 57) in general showed a higher cortisol concentration than males (m, n = 58). The age and gender groups combined show differences, with adult females (fa) having the highest and male yearlings (my) having the lowest cortisol levels. Different letters indicate a significant difference (e.g. significant higher cortisol levels in female adults compared to male adults, male yearlings, and female juvenile).

Female wild boar have significantly higher cortisol levels during drive hunts than male wild boar do (mean ± standard deviation of 469.65 ± 241.99 nmol/L compared to 353.67 ± 230.97 nmol/L for female and male, respectively; Kruskal–Wallis chi-squared = 11.491, df = 1, p = 6.993 × 10^–4^). Comparing age groups, there are no significant differences (mean ± standard deviation of 435.04 ± 233.52, 369.63 ± 273.89 and 418.19 ± 221.41 nmol/L for age group 0, 1 and 2, respectively; Kruskal–Wallis chi-squared = 4.326, df = 2, p-value = 0.115). Grouping sex and age classes, the lowest cortisol levels are found in male yearlings, whereas the highest value can be found in female adults (Fig. 4).

**Figure 5:**
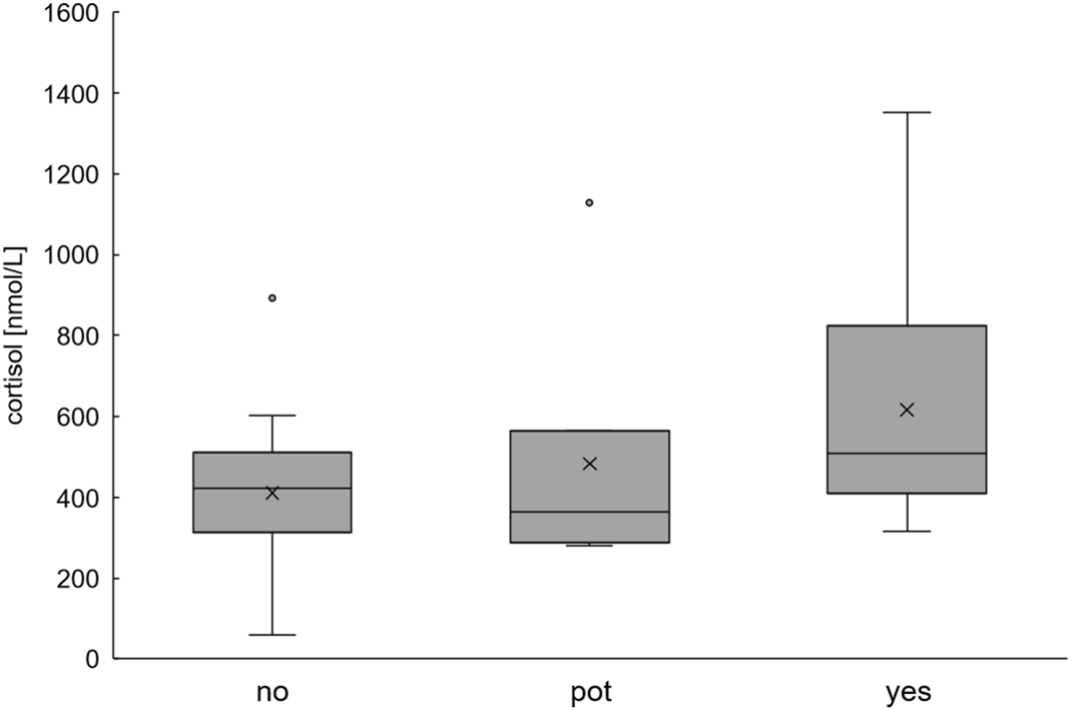
Concentration of cortisol levels of non-pregnant (no, n = 26), potentially pregnant (pot, a measurable number of Corpora lutea, n = 7) and pregnant (yes, development of fetuses, n = 14) wild boar. Although there is a noticeably increased cortisol level in pregnant wild boar, there are no significant differences among groups.

Non-pregnant and potentially pregnant wild boar show similar cortisol levels. After separating the cortisol values of definitely pregnant females, these animals were found to exhibit significantly higher cortisol levels, as compared to non-pregnant females (Kruskal–Wallis chi-squared = 4.1683, df = 1, p-value = 0.0412; Fig. 5). The number of foetuses does not have an influence on cortisol levels (Kruskal–Wallis chi-squared = 11.06, df = 7, p-value = 0.136).

## Discussion

We assumed that cortisol levels would naturally increase during drive hunts, as cortisol is one of the main hormones serving to increase energy release during a stress event^9^ and hunting is most likely to increase stress^21,32^. Our results show a very high variance with both highly increased, but also very low cortisol values. The variation could be caused by the great individuality of cortisol levels, which seems to be an important factor in stress related studies^10,49–52^.

Given the fact that we sampled after drive hunts, our results indeed show a high percentage of trauma cortisol levels (54%). Therefore, while the effect of hunting stress is present, the way we conduct drive hunts in Lower Saxony and most parts of Central Europe seems to be less stressful for wild boar than we expected (trauma cortisol levels in all samples). Although again with great variability, which could be again caused by the individuality but also small sample size in the respective hunting districts, there is no significant difference between hunting months and hunting area. This eliminates other factors, such as microclimate, habitat structure, and hunting intensity in the respective hunting areas to affect cortisol levels, as expected. It also gives the expression of no long-term effect of drive hunts on wild boar, as cortisol levels did not increase even after repeated drive hunts in the same area in the span of 2 or 2 months. An eventual customization/acclimatization to drive hunts resulting from the repetition of stressors^3,9,53^ such as drive hunts is also not noticeable. Still, it is important to keep in mind that we do not know the exact condition of the wild boar right before death. Differences in individual behaviour, e.g. the duration of them being chased, could cause differences in the level of stress the animal suffered from. Unfortunately, in this study the exact behaviour of the animal before the shot and time of death is unknown. For now, we must assume that all animals suffered from a similar amount of stress, as they all should have been alarmed by the start of the hunt.

The given differences between male and female wild boar, with females having an overall higher cortisol level compared to males (469.65 ± 241.99 nmol/L compared to 353.67 ± 230.97 nmol/L, respectively), during drive hunts were very prominent to note. Female wild boar form social groups consisting mostly of mothers with their offspring, and are, therefore considered to be matrilineal^18,54–56^. Male wild boar leave the group when reaching puberty^56^. Despite the benefits of association in female wild boar, costs like resource competition^57^ could lead to specific social pressure and might result in higher cortisol concentrations in females, as well as in group living male piglets (juvenile male). Differences in cortisol levels between sexes, as well as between social ranks, were also found in other species^58,59^, though the results seem to be contentious, depending on what assay was used. For instance, it was shown that growing pigs at the age of 4, 8, and 12 weeks exhibit no gender-based cortisol level differences^60^, similar to pigs until the weight of 104 ± 7 kg live body mass^61^. This supports our findings that there are no differences in the cortisol levels of female and male juvenile wild boar (Fig. 4). Other studies found significantly higher cortisol levels in male Yucatan minipigs, as compared to females^22^, and in barrows compared to gilts^62^.

Another explanation for the differences between sexes, other than the social structure of wild boar, is the general divergence of male and female wild boar based on metabolism and other processes, most importantly pregnancy, in female animals. Potentially pregnant wild boar showed similar cortisol levels to non-pregnant wild boar, and there was a higher cortisol level present in pregnant wild boar, although this was only significant when potentially pregnant animals were excluded (Fig. 5). This is also seen in humans, as cortisol increases exponentially throughout pregnancy and peaks at parturition^37,38^. The cortisol levels of potentially pregnant wild boar are, therefore, still comparable to those of non-pregnant individuals, because cortisol levels increase in the last months of gestation.

A correlation between cortisol levels and weight could not be found (Fig. 3), regardless of age and gender-related differences in weight. This is in contrast to findings showing relationships between cortisol levels and mass-specific metabolic rate, linked to body mass, in other mammals^4^.

To conclude, we were able to see elevated cortisol levels caused by drive hunts, but their effect was detracted by visible differences between age and sex groups, as well as the influence of pregnancy on cortisol levels, while differences between hunting months or hunting regions could not be found. Animal welfare is becoming increasingly important today. Stress, especially long-term stress, is said to negatively impact the health, reproduction, and longevity of wildlife, as well as influence the spread of diseases and affect host-parasite equilibrium^7^. Hunting and especially drive hunts are most likely to induce stress. However, our findings show not that big of an influence of drive hunts on chronic stress as initially expected. To strengthen our findings and look further into the influence of different types of hunting on stress in wild boar, as well as possible chronic stress triggers, we need to investigate the cortisol levels of more animals under different circumstances. Besides, different media for cortisol assaying, such as faeces, should be collected throughout the year to examine basal cortisol levels, to investigate seasonal or annual changes, and to explore possible chronic stress triggers such as hunting. This should not only be done in wild boar, but also other wildlife species.
